# Is malaria illness among young children a cause or a consequence of low socioeconomic status? evidence from the united Republic of Tanzania

**DOI:** 10.1186/1475-2875-11-161

**Published:** 2012-05-09

**Authors:** Marcia Caldas de Castro, Monica G Fisher

**Affiliations:** 1 Department of Global Health and Population, Harvard School of Public Health, 665 Huntington Avenue, Bldg, I, Room 1113, Boston, MA 02115, USA; 2 Agricultural and Resource Economics Department, Oregon State University, Ballard Extension Hall Room 218, Corvallis, OR 97331, USA

**Keywords:** Malaria, Poverty, Conceptual framework malaria-poverty causality

## Abstract

**Background:**

Malaria is commonly considered a disease of the poor, but there is very little evidence of a possible two-way causality in the association between malaria and poverty. Until now, limitations to examine that dual relationship were the availability of representative data on confirmed malaria cases, the use of a good proxy for poverty, and accounting for endogeneity in regression models.

**Methods:**

A simultaneous equation model was estimated with nationally representative data for Tanzania that included malaria parasite testing with RDTs for young children (six-59 months), and accounted for environmental variables assembled with the aid of GIS. A wealth index based on assets, access to utilities/infrastructure, and housing characteristics was used as a proxy for socioeconomic status. Model estimation was done with instrumental variables regression.

**Results:**

Results show that households with a child who tested positive for malaria at the time of the survey had a wealth index that was, on average, 1.9 units lower (*p*-value < 0.001), and that an increase in the wealth index did not reveal significant effects on malaria.

**Conclusion:**

If malaria is indeed a cause of poverty, as the findings of this study suggest, then malaria control activities, and particularly the current efforts to eliminate/eradicate malaria, are much more than just a public health policy, but also a poverty alleviation strategy. However, if poverty has no causal effect on malaria, then poverty alleviation policies should not be advertised as having the potential additional effect of reducing the prevalence of malaria.

## Background

**Figure 1:**
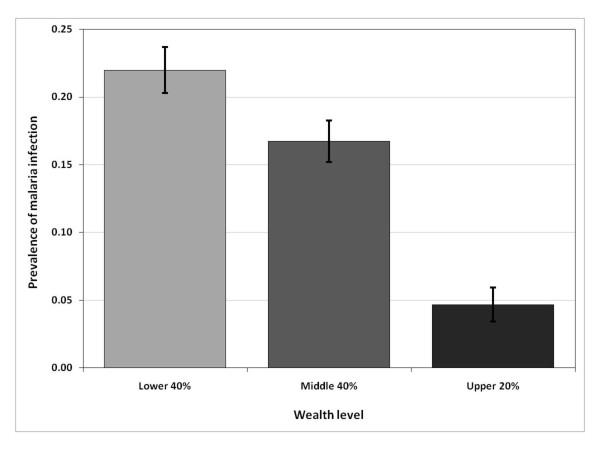
**Malaria prevalence among young children (six-59 months), by household wealth level, Tanzania, 2007/08.** The wealth variable is based on the THMIS wealth index, generated with principal components analysis. The categories poor, middle, and rich represent, respectively, the bottom 40%, next 40%, and upper 20% of the THMIS wealth index distribution.

Malaria is commonly considered a disease of poverty [1-3]. Recent Demographic and Health Survey (DHS) data for the United Republic of Tanzania (from now on referred to as Tanzania), illustrate this relationship at the individual child level (Figure 1). The observed negative correlation between malaria and socioeconomic status (SES) may indicate that malaria infections cause low SES (e.g. ill workers are less productive), or that poverty increases the risk of malaria transmission (e.g. the poor are less able to afford malaria preventative measures). Also, there may be incidental associations, such as, improvements in road infrastructure in a region could simultaneously increase household incomes (e.g. through improved market access) and reduce malaria incidence (e.g. through better access to health care facilities). Understanding whether the malaria-poverty correlation implies causality and, if it does, the direction of causality, has crucial implications for malaria control efforts, such as the Roll Back Malaria (RBM) and the President’s Malaria Initiative (PMI), and for the recent call for malaria elimination, with the ultimate goal of malaria eradication [4]. If a bi-directional link exits between malaria and SES, then (i) poverty alleviation policies (e.g. income redistribution, job creation, and educational investment) may have the side benefit of reducing malaria, becoming important tools to complement and maximize the impact of disease prevention and treatment efforts; and (ii) malaria control strategies may be beneficial for reducing poverty.

Worral, Basu, and Hanson [4] reviewed about 50 micro-level studies that examined whether SES influences the uptake of malaria prevention and treatment, and if malaria is more common among the poor. Results from the former tended to be in good qualitative agreement, and showed a positive correlation between SES and the use of preventative malaria measures such as insecticide-treated nets (ITNs). Regarding the incidence of malaria on the basis of poverty status, the magnitude and significance of results varied across studies, depending on how poverty and malaria were measured. This review effort, however, highlighted important caveats in the existing work [4]. First and foremost, studies assessed malaria incidence as self-reported fever episodes, which is very likely to overestimate the results since a significant percentage of fever episodes are not due to malaria infections [5-7]. Second, reviewed studies failed to account for possible two-way causality in the association between malaria and poverty; this methodological limitation suggests caution in comparison and interpretation of extant research.

Recent papers by Somi et al. [7,8] constitute the first attempts to address the research caveats mentioned above. The authors used laboratory-confirmed malaria morbidity data (parasitaemia based on microscopy), and accounted statistically for potential bi-directional malaria-poverty causality by using instrumental variable probit regression and propensity score matching. Data from 52 rural villages in south-eastern Tanzania, which comprised two Demographic Surveillance Sites (DSS), were used to construct a wealth index (proxy for SES), and to provide all other variables included in the model [8]. Results indicated that SES was negatively associated with malaria: a one-unit increase in the wealth index resulted in a 4% decrease in the prevalence of malaria infection. Malaria was also negatively associated with SES: an infection resulted in a reduction of 0.32 units in the wealth index [8].

In this paper, the aforementioned limitations are addressed, and three additional contributions to current knowledge are made. First, a formal conceptual framework of the two-way malaria-SES link is developed, utilizing a multi-disciplinary and multi-scale approach, which can be applied in other areas and can guide future empirical modelling. Existing work has relied on conceptual frameworks restricted to variables that are available for modelling exercises [8], and the lack of a formal conceptual framework hinders the comparability and replicability of studies to other settings [4]. Second, the bi-directional malaria-poverty causality is examined using a simultaneous equation model estimated with nationally representative DHS data for Tanzania that included malaria parasite testing for young children (six-59 months). This represents a major improvement to recent estimates that relied on data from a few rural districts [7,8]. Also, although a previous study utilized DHS data for 22 countries [9], it used self-reported information on fever in the two weeks preceding the survey as a proxy for malaria, which is not an accurate measure of the disease burden [10], and did not examine causality in both directions. Third, geo-referenced information is included in the model. Chima, Goodman, and Mills [6] have urged researchers to account for geographic variations in the relationship between malaria and poverty, but data limitations have often precluded spatially explicit analysis. The empirical analysis here presented benefits from the fact that the Tanzania DHS data contain such information. Among the geographic variations explored in the empirical modelling are climatic conditions, local infrastructure, proximity to putative sources of malaria transmission, and characteristics of the local- and human-made environment.

## Methods

### Study area

Tanzania is located in East Africa, along the shores of the Indian Ocean, between longitude 29^0^ and 41^0^ East, and latitude 1^0^ and 12^0^ South. Mainland Tanzania borders Kenya and Uganda (north); Rwanda, Burundi, and the Democratic Republic of Congo (west); Zambia and Malawi (south west); and Mozambique (south). Zanzibar lies off the eastern coast and is situated at approximately 30 km from the mainland. The country is divided into two unique rainfall patterns: unimodal and bimodal. The former has one marked rainfall season that often occurs between November/December and April, and is observed in the southern, south-western, central, and western areas of the country. Humidity is high between December and May. The bimodal pattern has two rainfall seasons, an intense one observed between March and May, and a milder one occurring between October and December. Humidity is normally high between March to June and November to December. Regardless of the rainfall pattern, temperatures are generally high between October and March.

According to the 2004 update of the Global Burden of Diseases (GBD) [9], 44% of the disease burden in Tanzania (as measured by disability-adjusted life years – DALYs) was due to infectious and parasitic diseases. Among those diseases, malaria carried the largest burden, 20%. Malaria is the main cause for inpatient and outpatient consultations and the major killer for children under five in Tanzania. Malaria transmission is stable perennial to stable seasonal in over 80% of the country and the remaining areas have unstable transmission prone to malaria epidemics [10]. As a result, all the country’s 42 million people are at risk of acquiring the disease.

Recent statistics [11] show that Tanzania had gross national income per capita of $1,200 (in 2007 purchasing power parity), and it ranked low on quality-of-life indicators such as life expectancy at birth (51 years males, 53 years females), the adult literacy rate (69%), and the under-five mortality rate (118 per thousand). As for malaria, the estimated number of clinical cases per year ranges between 14 and 18 million, and the estimated number of deaths is about 60,000 (approximately 80% of deaths are among children under five years of age) [11]. Malaria is the leading cause of outpatients, deaths of hospitalized people, and admissions to medical facilities of children less than five years of age. For these reasons, malaria is considered a major cause for low worker productivity among those between 15 and 55 years old, and a key impediment to human capital formation for people between five and 25 years of age. The disease is considered to be a formidable obstacle to foreign investment and economic development in Tanzania [12].

### DHS data

In 2007–08, a special DHS was carried out in Tanzania: the HIV/AIDS and Malaria Indicator Survey (THMIS). As part of the THMIS, blood samples were collected from children aged six-59 months. Malaria infections were assessed through the use of the Paracheck Pf^TM^ rapid diagnostic test (RDT). The THMIS interviewed the guardians of 7,502 children; 6,686 of these children were living with their guardian and therefore had information available on household and location characteristics. Only children aged six-59 months were eligible for malaria testing, and there were 5,955 children in this age group. The guardians of 5,627 of these children consented to the child being tested for malaria. There are missing values for 43 children for whom consent for malaria testing was received. Therefore, the THMIS has confirmed results of a malaria infection for 5,584 children aged six-59 months [12]. Dropping from the sample those children with missing values for wealth-related variables yielded a working sample of 5,547 young children. The survey was conducted from October 2007 to February 2008, is nationally representative, and key indicators can be calculated for urban and rural areas, and for regions [12]. Therefore, the survey comprises diverse settings: urban and rural, low and high malaria risk, poor and relatively wealthy.

As is usual practice in the DHS, the THMIS collected data on household and respondent characteristics, as well as information on malaria prevention and treatment outcomes. The survey also contains geographical information that allows for the assessment of spatial variations in the bi-directional malaria-poverty causality. First, it contains the centroids of the sample clusters, allowing for the assembling of varied cluster-level data. Second, the THMIS includes information on region of residence (there are 26 regions in Tanzania), facilitating the merging of various region-level information that characterizes the local environment (as described below) with individual/household data.

Individual-level THMIS variables utilized in this study include: (i) the result of the child’s malaria test (1 = child tested positive for malaria); (ii) age of the child tested for a malaria infection – categorized as six-23 months, 24–35 months, 36–47 months, and 48–59 months; (iii) a binary variable indicating if the mother of the child is engaged in farming activity; (iv) a continuous variable reporting the number of overnight out-of-town trips that the mother of the child made in the previous year; (v) a binary variable indicating that the child’s mother and/or the child’s father had secondary education or higher (generated from variables for the education level of the child’s mother and of the household head); and (vi) a binary variable indicating the use of an ITN by the child the night before the interview.

Household-level variables utilized are: (i) age of the head of the household in years – age squared was also included, in order to assess if the relationship between wealth and age increases as one ages; (ii) sex of the head of the household (1 = female); (iii) number of children aged under five years living in the household; (iv) number of children aged six-12 years living in the household; (v) number of adolescents aged 13–17 living in the household; (vi) number of adults aged 18–64 years living in the household; (vii) number of adults aged 65 or more years living in the household; (viii) a binary variable indicating if the house received indoor residual spraying (IRS) in the previous year; (ix) a binary variable indicating if the household is located in a rural area; (x) a binary variable indicating if the house has improved roof material (iron sheet, concrete, tiles or asbestos); (xi) a binary variable indicating if the house has improved wall material (brick, wood/timber, cement, stones or iron/metal); (xii) physical assets owned by the household (car, motorbike, bicycle, fridge, television, radio and mobile phone); (xiii) source of water; (xiv) type of toilet; (xv) access to electricity; (xvi) number of rooms per household member; and (xvii) type of flooring material in the house. Although the THMIS has a variable to measure the general health status of the household head, and this variable is expected to be important to household wealth, its inclusion would reduce the sample by more than 10% due to missing values. Therefore, the variable was not considered in the model.

Based on household-level variables (xii)-(xvii), a wealth index was created using principal component analysis (PCA), as proposed by Filmer and Pritchett [13] and Vyas and Kumanarayake [14]. The first principal component was used to determine weighting factors for each variable measuring wealth and to define the wealth index. Although the THMIS does provide a wealth index calculated using the same procedure, the index includes bed net ownership, and type of roof and walls used in the house. These variables, however, are explanatory factors of the prevalence of malaria infection, and therefore should not be included in the wealth index.

To control for the seasonality of malaria transmission, the study utilized binary variables indicating the month between October 2007 and February 2008 when the household interview and malaria testing took place. Finally, cluster-level variables collected by the THMIS included in the analysis are: (i) average elevation; and (ii) distance from the cluster centroid to the nearest health facility (km).

### Regional data on the local environment

In order to better account for potential impacts of the local environment, data were assembled from varied sources with the aid of geographical information systems (GIS). Table 1 summarizes all the environmental data gathered for the analysis, and the sources from which they were obtained. Data were treated to reflect region-level characteristics, which could then be merged with the THMIS data.

**Table 1:** Environmental information assembled with the aid of GIS

**Variable**	**Source**
Rainfall	Africa Data Dissemination Service (ADDS) http://earlywarning.usgs.gov/fews/africa/index.php
Temperature	WorldClim – Global Climatic Data http://www.worldclim.com/current#ESRI%20grids
Road network	Africover http://www.africover.org/ Vector Map (VMap) level 0 http://www.mapability.com/ index1.html?http&&&www.mapability.com/info/ vmap0_index.html
Elevation	Consortium for Spatial Information (CGIAR-CSI) http://srtm.csi.cgiar.org/
Land Cover, Rivers	Africover http://www.africover.org/
Lakes	FAO Aquaculture Management and Conservation Service http://www.fao.org/geonetwork/srv/en/

Rainfall data were derived from satellite-based estimates [15] available by dekads (periods of roughly 10 days). Different ways to summarize rainfall were tried, in order to test which variable would be able to better capture the relationship between precipitation and malaria [16,17]. Thus, the following variables were constructed at the regional level: (i) total and variance of rainfall in the dry, rainy, and agricultural seasons, as well as deviations of the total from a short-term (2003–2008) mean; (ii) total amount of rainfall by month, as well as deviations of monthly rainfall from the short-term mean; and (iii) proportional difference between the rainfall in the survey period and two months prior to the survey period and the short-term mean (calculated as the rainfall for August 2007 to February 2008, subtracted by the short-term mean for August to February, and divided by the short-term mean for these months).

The road network layer was rasterized in ArcMap (ESRI, Redlands, CA, USA) to allow for the calculation of a road density indicator for each region (percentage of the region’s area utilized as roads). Since there is no standard width for roads, a 10 m-wide road was considered based on the current recommendations for construction of new roads in the country. A similar procedure was utilized to obtain the percentage of the region’s area covered by rivers, assuming an average width of 3 m. In addition, the different types of land use were summarized by region, and the percentage of the region being used for agricultural cultivation was included in the analysis. Also, the coefficient of variation of the slope was calculated by region for inclusion in the analysis. Finally, a layer with major lakes in Tanzania was utilized to calculate the distance from each cluster centroid to the nearest lake (km).

### Conceptual framework

A household’s SES is expected to impact malaria incidence among its members primarily because limited economic resources reduce the uptake of malaria preventative and/or curative measures, such as use of anti-malarial drugs, regular adoption of mosquito avoidance measures, and search for health care [5]. This is highly plausible since those measures have substantial direct costs. In sub-Saharan Africa, households spend as much as $180 per year (1999 US dollars) on such measures, which represents a sizeable share of household income [6]. In the opposite direction, it is anticipated that malaria illness reduces a household’s potential to accumulate wealth in at least three ways. First, ill health reduces labour supply and productivity, and the resultant reduction in household income makes saving difficult [6,14-16]. The time lost per malaria episode for a sick adult ranges, on average, from one to five days, and the same amount of time is lost to work when adults care for a sick child with malaria [6]. Second, malaria imposes health costs and, in the absence of formal health insurance markets, individuals may cover such costs by drawing down their savings, selling physical assets, or borrowing money [6,14]. Third, malaria may induce households to change their productive activities *ex ante*, and such adaptation may come at a cost to wealth accumulation [6,17,18]. Thus, it is hypothesized that a vicious circle of malaria illness and low SES exists, and this analysis aims to study and quantify these relationships empirically.

**Figure 2:**
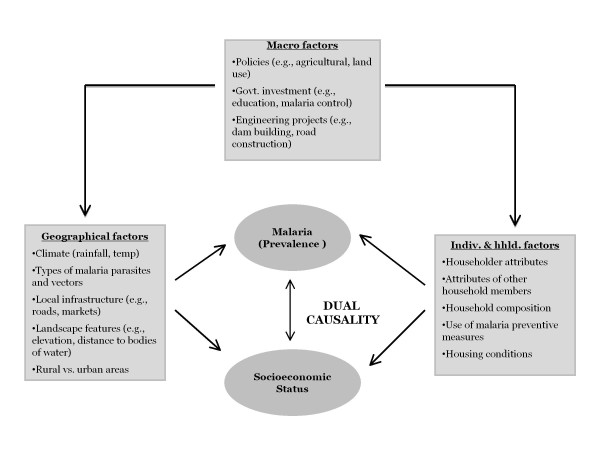
Conceptual framework of the bi-directional malaria-poverty causality.

The conceptual framework is proposed based on literature review of factors that explain malaria prevalence and SES (Figure 2). The framework provides a foundation for studying the two-way relationship between malaria and SES, proxied by wealth, as explained later in this section. To understand and measure bi-directional malaria-poverty causality, the conceptual framework (Figure 2) accounts for numerous factors that interact and contribute to transmission of malaria and to poverty. These factors are grouped in three categories: (i) individual and household; (ii) geographical; and (iii) macro. The first includes: (a) individual characteristics: genetic immunity; acquired immunity; migratory pattern; age; economic activity; education; and cultural beliefs; (b) household composition: number of household members by age category; (c) housing conditions: quality of walls, floor, and ceiling; screening; and presence of eaves; (d) use of malaria preventative measures: ITNs, repellents, anti-malarials, IRS; and (e) knowledge about malaria transmission, prevention and treatment. Geographical factors encompass: (a) natural environment: temperature, humidity and rainfall; soil quality; elevation/slope; land cover; hydrography; (b) human-made environment: land use and land change; local infrastructure – roads, sanitation, clean water; access to markets; urban or rural condition; and (c) types of malaria parasites and vectors. Lastly, macro factors describe: (a) governmental policies and investment, such as public health interventions, educational programmes and agricultural incentives; and (b) implementation of new infrastructure projects, such as roads, railways and hydroelectric dams. It is assumed that macro factors are distal determinants of both malaria and SES, since their impacts are likely to act through individual, household and geographical factors. More details on each of the factors are provided in Additional file 1.

Turning to wealth, the conceptual framework assumes household wealth holdings provide a useful metric for economic well-being. In the poverty literature, SES is typically measured in terms of expenditure or income. Such information is unavailable in the DHS. The decision to use a wealth-based SES measure, however, is neither unprecedented [7,13,18,19] nor purely for practical reasons. A recent comparison of SES measures for multivariate analysis of the socioeconomic gradient of malaria prevalence found that a wealth-based index is a useful alternative to the usual consumption measure [7]. In addition, wealth provides a more complete picture of household living standards than income. Wealth provides a household with economic stability, because households with liquid assets are better able to endure income shortfalls. A household experiencing temporary low income due to job loss of a household member could be classified as income poor. In fact, such a household may not experience economic hardship if liquid assets are available to smooth consumption over income fluctuations. Another key role of assets is in providing a foundation for risk taking that leads to resource accumulation over time [20]. For instance, household savings can be used to start up a business or invest in a child’s education. Thus, while a lack of income means that people struggle to get by, a lack of assets can prevent them from getting ahead. More details are provided in Additional file 1.

### Empirical model

To examine whether the observed association between malaria prevalence (*M*) and household wealth (*W*) implies causality, a simultaneous equation model was estimated, as summarized by equations (1) and (2)

(1) $$M_{\mathrm{ij}}=\alpha_{0}+\alpha_{1}W_{\mathrm{ij}}+\alpha_{2}X_{\mathrm{ij}}^{M}+\alpha_{3}G_{j}^{M}+\alpha_{4}S+\varepsilon_{\mathrm{ij}}^{M}$$

(2) $$W_{\mathrm{ij}}=\beta_{0}+\beta_{1}M_{\mathrm{ij}}+\beta_{2}X_{\mathrm{ij}}^{W}+\beta_{3}G_{j}^{W}+\varepsilon_{\mathrm{ij}}^{W}$$

In both equations, *i* and *j* index individuals (children aged six-59 months) and regions, respectively; ***X*** is a vector of individual- and household-level factors; ***G*** is a vector of geographic determinants; ***S*** represents a set of binary variables for the month of interview; *α* and *β* are coefficients to be estimated; and *ϵ* is the error term. The parameters of primary interest to this study are *a*_1_, which indicates the direction and magnitude of the link from wealth to malaria prevalence, and *b*_1_, which indicates the direction and magnitude of the link from malaria prevalence to wealth. Table 2 provides definitions and descriptive statistics for all variables included in the model.

**Table 2:** Descriptive statistics for variables included in the empirical model (*n* = 5,547)^1^

**Variable label**	**Variable definition and equation in which the variable is included - Malaria (M) and/or Wealth (W)**		**Mean or proportion^2^**	**Range**	
				**Min**	**Max**
*Dependent variables*					
mal_yn	Child tested positive for malaria (0/1)	M, W	0.170	0	1
wealth	PCA wealth index	M, W	−0.291	−1.592	10.566
*Individual-level explanatory variables*					
age1_yn	Child aged 6–23 months	reference^3^	0.384	0	1
age2_yn	Child aged 24–35 months	M	0.221	0	1
age3_yn	Child aged 36–47 months	M	0.189	0	1
age4_yn	Child aged 48–59 months	M	0.207	0	1
farming	Farming is the main occupation of mother	M, W	0.719	0	1
trips	Trips outside the community taken by the child’s mother in the last 12 months	M	0.638	0	15
education	Child’s mother and/or father has a secondary school education or higher	M, W	0.086	0	1
itn_yn	Child slept under an ITN the night before (0/1)	M	0.117	0	1
*Household-level explanatory variables*					
agehd	Householder’s age (years)	W	41.377	16	95
femhd_yn	Householder is female (0/1)	W	0.154	0	1
ychild	Number of young children (0–5 years)	W	2.198	0	12
child	Number of children (6–12 years)	W	1.425	0	9
teen	Number of teenagers (13–17 years)	W	0.672	0	7
adult	Number of adults (18–64 years)	W	2.776	0	13
elder	Number of elders (65 years +)	W	0.159	0	4
improof_yn	House with improved roofing (0/1)	M	0.471	0	1
impwall_yn	House with improved walls (0/1)	M	0.702	0	1
irs_yn	Indoor residual spraying last year (0/1)	M	0.037	0	1
rural_yn	Household resides in a rural area (0/1)	M, W	0.833	0	1
*Month-of-interview explanatory variables*					
oct_yn	Interview took place in October	reference ^3^	0.094	0	1
nov_yn	Interview took place in November	M	0.267	0	1
dec_yn	Interview took place in December	M	0.260	0	1
jan_yn	Interview took place in January	M	0.334	0	1
feb_yn	Interview took place in February	M	0.044	0	1
*Cluster-level explanatory variables*					
elevation	Average elevation (1,000 m)	M, W	1.094	0.003	5.750
disthfac	Distance to nearest health facility (km)	M, W	5.013	0	56
distlake	Distance to the nearest lake (km)	M, W	60.792	0	258.460
*Region-level explanatory variables*					
rain_dev	Rainfall (mm) Aug 2007-Feb 2008 minus the short-term mean (2003–08) for Aug-Feb divided by the short term mean	M, W	0.394	−0.520	0.631
roads10	% of area covered by 10 m-wide roads	M, W	0.0008	0.0004	0.0027
rivers3	% of area covered by 3 m-wide roads	M, W	0.034	0	0.056
farmland	Proportion of area under agriculture	M, W	0.398	0.172	0.867
slope_cv	Slope of the terrain (coeff. of variation)	M, W	1.234	0.630	1.560

^1^ For most variables the sample size is 5,547; it can be slightly less due to missing values.

^2^ Means, proportions, minimums, and maximums are weighted using the THMIS household weights.

^3^ Excluded from the regressions - reference category with which coefficients for other ages are compared.

A key estimation issue is simultaneity bias, due to joint determination of *M* and *W*. Simultaneous equation models generally violate a standard assumption of the classical linear regression model, which states that all explanatory variables should be uncorrelated with the error term [62]. To illustrate that this assumption is unlikely to hold, consider the situation where ***X***^***M***^ in equation (1) increases in a given time period. *Ceteris paribus*, this would be associated with an increase in *M*. Turning to equation (2), it is apparent that an increase in *M* will, all else being equal, be associated with an increase in *W*. This suggests that *e*^*M*^ and *W* increase together; that is, there is correlation between an explanatory variable and the error term. If the simultaneous model was estimated using ordinary least squares (OLS), the correlation between error terms and endogenous explanatory variables would result in biased estimates of *a*_*1*_ and *b*_*1*_. To demonstrate the source of bias, consider *a*_*1*_, which is supposed to be the effect of *W* on *M* holding other factors constant. In a simultaneous model estimated with OLS, *a*_*1*_ instead measures some combination of the effects of *W* and *M* on each other due to joint determination. Therefore, to obtain consistent estimates of the dual causality between malaria and wealth, the model described by equations (1) and (2) were estimated using instrumental variables (IV) regression [62]. In this approach, the basic strategy to deal with simultaneity bias is to find proxy variables for the endogenous explanatory variables *M* and *W*. The key to the IV approach is finding appropriate “identifying instruments” to include in the first-stage regression. A valid identifying instrument is a variable that is highly correlated with the endogenous variable, and uncorrelated with the error term. As a test for IV validity, pair-wise correlations were calculated, and assessed the significance of the selected IVs in the model. The wealth equation was identified using the following IVs: binary variables for the child’s age and binary variables for the month of interview. These IVs are expected to be highly associated with malaria incidence but to have no direct association with household wealth. Note that the variables for bed net, IRS, improved roof, improved walls, and geographic mobility of the child’s mother were excluded from the wealth equation due to a concern that these variables are endogenous to wealth and could thereby result in biased parameter estimates. The malaria equation (1) was identified with the following instruments: age and gender of the household head, and household composition variables. The household composition variables are numbers of young children (less than five years of age), children aged five to 12 years, teenagers aged 13–17 years, adults aged 18–64 years, and elderly aged 65 years and over. These IVs are expected to be highly correlated with household wealth but to not directly influence malaria transmission.

To explain the IV approach, the following steps were taken to estimate the effect of malaria prevalence on household wealth. The first step was to re-write structural equation (1) as a reduced form in which malaria is expressed as a function of all exogenous variables in the simultaneous equation system plus an error term. This was done by substitution of equation (2) into equation (1) and collecting terms, to arrive at equation (3). A re-labelling of the parameters yielded the reduced-form equation (4).

(3) $$\begin{array}{l} M_{\mathrm{ij}}=\left(\frac{\alpha_{0}+\alpha_{1}\beta_{0}}{1-\alpha_{1}\beta_{1}}\right)+\left(\frac{\alpha_{2}}{1-\alpha_{1}\beta_{1}}\right)X_{\mathrm{ij}}^{M}+\left(\frac{\alpha_{3}}{1-\alpha_{1}\beta_{1}}\right)G_{j}^{M}+\left(\frac{\alpha_{1}\beta_{2}}{1-\alpha_{1}\beta_{1}}\right)X_{\mathrm{ij}}^{W}\\ {{}_{}}_{}{{}_{}}_{}{}_{}+\left(\frac{\alpha_{1}\beta_{3}}{1-\alpha_{1}\beta_{1}}\right)G_{j}^{W}+\left(\frac{\alpha_{4}}{1-\alpha_{1}\beta_{1}}\right)S+\left(\frac{\alpha_{1}\varepsilon_{\mathrm{ij}}^{W}+\varepsilon_{\mathrm{ij}}^{M}}{1-\alpha_{1}\beta_{1}}\right) \end{array}$$

(4) $$M_{\mathrm{ij}}=\delta_{0}+\delta_{1}X_{\mathrm{ij}}^{M}+\delta_{2}G_{j}^{M}+\delta_{3}X_{\mathrm{ij}}^{W}+\delta_{4}G_{j}^{W}+\delta_{5}S+\upsilon_{\mathrm{ij}}^{M}$$

The next step in the IV approach was to estimate reduced-form equation (4). The third step was to use the first-stage regression results to obtain the predicted value of *M* (prevalence of malaria infection). The final step in the IV approach was to estimate structural equation (2). In this second-stage regression, the predicted value of *M* from the first-stage probit regression was used as the malaria explanatory variable. By substituting *predicted* malaria prevalence for *observed* malaria prevalence, we presumably eliminated the correlation between the explanatory variable *M* and the error term *e*^*W*^. To account for the binary nature of the malaria variable *M*, a treatment regression approach for the first- and second-stage regression models was used; the treatment in this case was having malaria. The treatment regression model is estimated with maximum likelihood estimation. The IV approach for estimating the effect of wealth on malaria is similar to that described above, but the model is an IV probit model since the first-stage model has a continuous dependent variable while the second-stage model has a binary dependent variable. All model estimations were conducted using Stata version 11 (Stata Corp.; College Station, TX, USA).

## Results

### Wealth index

Table 3 presents the results for tests of internal coherence of the wealth index. The table displays, by wealth groupings, the averages for variables measuring asset ownership, access to utilities and infrastructure, and housing conditions. In general, the results indicate sizeable differences across wealth groups, and these differences were in the direction that would be expected if the wealth index provided a good measure of SES. For example, while none of the poorest households owned a mobile phone, 33% and 89% of the middle and richest households did, respectively. The percentage of households that had access to an improved source of water also increased by wealth group. The finding that the middle of the wealth distribution had the highest average bike ownership suggests that as wealth increases households were initially more likely to own a bicycle but at a certain wealth level bicycle ownership declined. This is likely explained by wealthier households preferring to purchase a motorbike or a car rather than a bike.

**Table 3:** Checks for internal consistency of the wealth index, Tanzania, 2007–08 (*n* = 5,547)

**Variable**	**Wealth category**		
	**Poorest 40%**	**Middle 40%**	**Richest 20%**
*Asset ownership (proportion)*			
Has car	0	0	0.058
Has motorcycle	0	0.002	0.076
Has bicycle	0.452	0.605	0.460
Has fridge	0	0	0.201
Has television	0	0	0.410
Has radio	0.345	0.792	0.893
Has mobile phone	0	0.333	0.887
*Access to utilities/infrastructure (proportion)*			
Home has electricity	0	0.003	0.491
Has improved source of drinking water	0.233	0.698	0.858
Has improved toilet	0	0.010	0.370
*Housing characteristics*			
Rooms for sleeping per person (mean)	0.319	0.399	0.433
Floor of home is finished (proportion)	0	0.193	0.937

### Does malaria illness among young children contribute to low household wealth?

The wealth model suggests that malaria illness among young children (six-59 months) was a contributing factor for low household wealth in Tanzania (Table 4). The correlation between malaria and the IVs used in the model were statistically insignificant at standard test levels (5%) or had coefficients close to zero, and most of the IVs were highly significant in the wealth equation. These results support the validity of the IVs used in the malaria model. Results shown in Table 4 indicate that the malaria variable, which was predicted from a first-stage, reduced-form regression, was highly statistically significant. Controlling for other factors that influence wealth, households that had a child who tested positive for malaria at the time of the survey had a wealth index that was, on average, 1.9 units lower (*p*-value < 0.001). To put this figure in perspective, note that the standard deviation for the wealth index is about 2, which suggests that malaria among young children had a large negative effect on household wealth.

**Table 4:** Treatment regression results for the wealth index model (*n* = 5,340) ^1^

**Variable**	**Coefficient ^2^**	**95% Confidence interval ^3^**	
		**Lower CI**	**Upper CI**
constant	* 0.779	0.073	1.485
mal_yn	* -1.902	−2.076	−1.729
ageh	0.010	−0.013	0.032
agesq	−0.0002	−0.0005	0.00003
femaleh_yn	* -0.204	−0.344	−0.065
ychild	* -0.078	−0.128	−0.028
child	−0.023	−0.070	0.025
teen	* 0.096	0.019	0.174
adult	* 0.138	0.086	0.189
elder	* 0.245	0.068	0.422
farming	* -0.605	−0.731	−0.479
education	* 1.375	1.151	1.599
rural	* -1.210	−1.434	−0.986
elevation	* -0.223	−0.312	−0.133
HF_dist	* -0.014	−0.022	−0.007
lake_dist	−0.0003	−0.001	0.001
rain_dev	* 0.595	0.341	0.850
roads10	* 521.038	323.195	718.880
rivers3	0.455	−3.333	4.242
farmland	−0.001	−0.353	0.350
slope_cv	−0.027	−0.311	0.256

^1^ The sample size for Tables 2 and 3 differs from the sample size for Tables 4 and 5 because some of the explanatory variables had missing values leading to additional reductions in sample size for the regressions.

^2*^ Indicates statistical significance at the 0.05 significance level or better.

^3^ The estimation of standard errors was adjusted for clustering on households to account for possible non-independence of observations within households. It is expected that children in the same household are similar to each other on account of shared genetics and/or home environment.

Results for the control variables provide an indication of how well the model fits the data. Twelve of the 19 controls were statistically significant at standard test levels and findings for these variables conform to prior expectations, as previously described in the proposed conceptual framework. Results for household-level variables indicate that household wealth was negatively associated with female headship, number of young children, and agricultural occupation of the child’s mother. Household wealth was positively associated with number of household members aged 13 and above, and secondary education of the child’s mother and/or father. Households had lower wealth if they were located at higher elevations, had poor market access (as measured by 10 m-wide road density and distance to health facility), and were in a rural area. Higher rainfall during the survey period compared to the short-term mean for 2003–2008 was correlated to higher wealth, which is as expected in a country where the majority of the population earns a living from rain-fed agriculture.

### Does low household wealth increase the risk of malaria illness among young children?

Table 5 presents the results of the malaria model. Lack of significant correlations between the IVs and wealth, and the fact that most IVs were significant in the malaria equation provide evidence of the validity of the instruments used. Model results indicate that malaria prevalence among young children was unrelated to the household’s wealth position. The coefficient of the wealth index had the anticipated negative sign, but it was not statistically significant (*p*-value = 0.677).

**Table 5:** Instrumental variables probit regression results for the malaria model (*n* = 5,340)

**Variable**	**Marginal effect ^1^**	**95% Confidence interval ^2^**	
		**Lower CI**	**Upper CI**
wealth (predicted)	−0.007	−0.041	0.027
age2_yn	* 0.038	0.019	0.058
age3_yn	* 0.034	0.014	0.054
age4_yn	* 0.052	0.030	0.073
farming	0.009	−0.014	0.032
trips	0.0002	−0.006	0.006
education	−0.002	−0.047	0.043
itn_yn	* -0.016	−0.031	−0.001
improof_yn	−0.015	−0.043	0.014
impwall_yn	* -0.047	−0.072	−0.021
irs_yn	* -0.036	−0.070	−0.001
rural_yn	* 0.035	0.005	0.066
nov_yn	0.023	−0.009	0.056
dec_yn	0.032	−0.002	0.066
jan_yn	* 0.043	0.007	0.079
feb_yn	0.042	−0.010	0.093
elevation	* -0.051	−0.069	−0.033
HF_dist	* 0.001	0.00004	0.002
lake_dist	* -0.0002	−0.0004	−0.0001
rain_dev	* 0.229	0.185	0.272
roads10	* -115.882	−150.108	−81.655
rivers3	* 0.673	0.110	1.236
farmland	* 0.193	0.138	0.247
slope_cv	0.012	−0.034	0.058

^1*^ Indicates statistical significance at the 0.05 significance level or better.

^2^ The estimation of standard errors was adjusted for clustering on households to account for possible non-independence of observations within households. It is expected that children in the same household are similar to each other on account of shared genetics and/or home environment.

Turning to the control variables for the full model, children less than two years of age had lower malaria prevalence than children between the ages of two and four years. Children were less likely to have malaria if they lived at higher elevations, if they lived in proximity to a health facility, if road density was high, if they slept under an ITN the night prior to the interview, and if they lived in houses where IRS was done in the year before the survey. Malaria prevalence was higher if rainfall during the survey period was greater than the mean value observed during 2003–2008. Living close to a lake, river, agricultural field, or in a rural area, was found to be linked to higher malaria prevalence. Finally, children were more likely to test positive for malaria in January 2008 compared to October 2007. This finding is not unexpected given that rainfall levels were, on average across the 26 Tanzania regions during the survey period, 630 mm higher in January than in October.

## Discussion

This paper proposed a conceptual framework of the bi-directional link between malaria and SES, utilizing a multi-disciplinary and multi-scale approach. The framework presented a comprehensive picture of mechanisms through which malaria and SES may impact each other, and guided the selection of variables included in the empirical model here estimated. Many of the factors listed in the framework were not available in the THMIS and, therefore, the empirical model was as comprehensive as the availability of data allowed. Yet, the inclusion of environmental variables generated with the aid of GIS was an important contribution to the analysis, and facilitated controlling for potential impacts of the natural and human-made environment on the prevalence of malaria infections.

The bi-direction association between malaria and SES was appraised with nationally representative data for Tanzania that assessed malaria infections based on RDTs, accounting for environmental variables assembled with the aid of GIS. Results show that households with a child who tested positive for malaria at the time of the survey had a wealth index that was, on average, 1.9 units lower (*p*-value < 0.001), and that an increase in the wealth index did not reveal significant effects on malaria. These results differ from Somi et al. [8], who reported that malaria was negatively associated with SES, and that SES was also negatively associated malaria. However, results here presented agree with the bulk of the literature that finds no statistically significant association between SES and malaria illness [4], although the latter literature does find that SES has a positive influence on malaria prevention and treatment seeking. Also, the results here presented are specific to children aged six-59 months, while Somi et al. [8] did not restrict their analysis to this age group.

Findings of the present study implicating malaria incidence as a cause of poverty, and those presented by Somi et al. [8], have important policy implications. If malaria is indeed a cause of poverty, then one could argue that malaria control activities, and particularly the current efforts to eliminate/eradicate malaria, are much more than just a public health policy, but also a poverty alleviation strategy. The lack of an effect of SES on malaria also has important policy implications. If poverty has no causal effect on malaria, then poverty alleviation policies should not be advertised as having the potential additional effect of reducing the prevalence of malaria. For example, recent studies showed evidence that microfinance institutions can be used to effectively deliver malaria knowledge to those communities benefiting from loans, and that had a significant impact on prevalence levels [21,22]. Yet, there is no evidence that poverty reduction resulting from microfinance programmes can solely result in lower malaria transmission.

This study has some limitations. First, the validity of the proposed identifying instruments could be disputed. However, supporting empirical evidence of their appropriateness was presented in the Results section. Second, the THMIS data are cross-sectional and observational, which limits the ability to infer causality. Future research on malaria-poverty dual causality should use longitudinal data and take advantage of natural experiments, to the extent that this is possible. Third, the proxy for SES was a wealth index computed through PCA, based on asset ownership, access to services/infrastructure, and housing characteristics. The THMIS data did not include information on current value, purchase price, or vintage of assets. These data omissions posed some complications for assigning a monetary value to the household’s stock of wealth. As a result, the wealth index is a relative measure of well-being. Therefore, results cannot be used to estimate the magnitude of the reduction in household wealth resulting from malaria illness among children. Datasets with other measures of economic well-being, such as income and consumption, would facilitate estimating some of the economic costs of malaria illness among young children. Fourth, the clusters used in the THMIS do not have spatial boundaries, due to confidentiality issues. Therefore, while cluster centroids are provided by the THMIS, cluster-level environmental data cannot be assigned, and the only geographical scale that could be used was the region. Finally, the effect of malaria illness among young children on household SES is likely to be of a different magnitude from the effect among working adult members. On the one hand, malaria episodes among young children should be more severe and therefore involve greater costs for treatment. On the other hand, malaria illness among working adults should have more of an impact on household income via reductions in labour supply and labor productivity. Further research is now being conducted, using other DHS data that will enable comparison of malaria-SES effects for women (including pregnant women) and young children.

## Competing interests

The authors declare that they have no competing interests.

## Authors' contributions

MCC and MGF designed the study, proposed the conceptual framework, developed the empirical model, gathered and analysed the data, drafted the manuscript, and equally share the authorship of the paper. All authors read and approved the final manuscript.

## Supplementary Material

Additional file 1 Details on the conceptual framework [23-57]. Click here for file
