# Supplementary material for: Is malaria illness among young children a cause or a consequence of low socioeconomic status? evidence from the united Republic of Tanzania
Source: Malar J. 2012 May 9;11:161. doi: 10.1186/1475-2875-11-161 (PMC3439375; doi:10.1186/1475-2875-11-161)
Supplement: Additional file 1 — Details on the conceptual framework [23-57]. [file 1475-2875-11-161-S1.doc]

**Is malaria illness among young children a cause or a consequence of low socioeconomic status? Evidence from the United Republic of Tanzania**

**Details on the conceptual framework**

Individual and household factors that influence malaria transmission often alter the level of exposure to an infection. For example, with regard to immunity, genetic susceptibility to malaria relates to the presence of sickle cell trait, beta-thalassaemia or Duffy blood factor [19,20]. Also, exposure to malaria over several years can provide acquired immunity, attenuating the most severe symptoms of the disease and resulting in asymptomatic cases; however, immunity is lost when exposure to malaria stops [1-2]. This is reflected by the distinctive age pattern of malaria transmission in sub-Saharan Africa: children below five years of age are under higher risk of morbidity and mortality [3], although the risk is lowest for children less than six months of age due to immunity inherited from the mother [4]. In addition, immunity to malaria among pregnant women is temporarily impaired, and the disease is an important cause of still births and infant mortality [5]. Exposure to malaria is also impacted by the type of occupational activity and by housing conditions. In addition, human migration influences malaria incidence through several mechanisms [6-8], such as: (i) seasonal movements to avoid the peak of transmission [9]; (ii) flow of non-immune individuals to new settlement areas close to forests [10]; (iii) involuntary movements due to war, persecution and natural disasters [11]; and (iv) high human mobility of infected individuals, who act as carriers of the parasite [12]. Housing characteristics (e.g. screening, roof materials and open eaves) indicate the level of protection residents have against indoor mosquito biting [13-14], and the use of selected preventative measures contribute to reduce human-vector exposure [5].

With regard to geographical factors, local climatic conditions (rainfall, temperature and humidity) affect the density of mosquitoes and the seasonality of transmission [2, 15-16]. Very high temperatures may also increase human-vector contact through individual behaviour change, by discouraging bed net use and encouraging outdoor sleeping [17]. Also, the types of malaria parasites, the prevalence of certain vector species, and the development of resistance to anti-malarials [38,39] all vary across space. The biology of vector and parasites play an important role in malaria transmission to the extent that it can increase human exposure and limit the selection of control interventions – e.g. an outdoor biting pattern limits the use of IRS and bed nets [12]. Local infrastructure (e.g. roads and health facilities) influences malaria incidence through effects on access to malaria prevention and treatment, and via the implementation of systematic and spatially targeted malaria control interventions (e.g. distribution of bed nets, IRS, and larviciding). Landscape features, such as topography, land cover and hydrography, may increase or decrease transmission levels. While elevation is a good proxy for temperature, the slope tends to influence the abundance of mosquito breeding habitats depending on the terrain conditions. Large variations in slope may facilitate the accumulation of water bodies early and late in the rainy season [18]. Proximity to putative sources of malaria transmission (e.g. river beds, drains, agricultural fields and bodies of stagnant water) may increase the risk of transmission depending on vector breeding and feeding preferences and the force of transmission [19-20]. Agricultural fields, particularly irrigated rice fields, have been linked to high malaria transmission [21-22]. Conversion of forest to farmland opens up new areas that may offer ideal conditions for mosquito breeding and reduce the proximity between human and mosquito [12, 23]. The influence of urbanization on malaria transmission can be three-fold. First, it might contribute to a reduction in the number of places that could potentially act as Anopheles breeding sites given the large extent of built-up areas and drainage [24]. Second, the initial process of urban expansion in the periphery of the city is most often characterized by fast developing unplanned settlements, lacking basic infrastructure and, therefore, accompanied by increases in Anopheles larval habitats [25]. Finally, high levels of population density, such as currently observed in urban agglomerations, ultimately contribute to reduce the intensity of malaria transmission [26-28].

Macro-level variables have an indirect effect on malaria prevalence via their impacts on local geographical conditions and on individual/household characteristics. For example, government investment in malaria control efforts affects the level of knowledge about malaria and access to malaria preventative and treatment methods and, subsequently, is likely to reduce malaria prevalence. The construction of dams, however, has historically resulted in increased malaria transmission [29-31].

The relationship between wealth and the factors discussed above are related to the primary reasons people save [32]. A main motivation for saving is the life-cycle motive, whereby wealth accumulation provides a means to smooth consumption across the life cycle, with individuals typically having positive net savings during their working years and drawing down their wealth upon retirement [33]. Therefore, according to this theory, all else being equal, household wealth will have an inverse-U relationship with the age of the householder. Life-cycle models also suggest that expenditure on children may serve as a substitute for savings, if parents rely on children for support in old age, which is common in developing countries. The implication is that there should be a negative correlation between number of children and household savings [56]. The savings motive is also influenced by an individual’s time horizon, since the act of saving requires giving up consumption today for returns in the future. People in poor health who expect to die young should have relatively short time horizons and thereby less incentive to save [58]. Therefore, important individual/household factors related to wealth are thus the householder’s age and her/his general health status, and the number of children in the household.

Another important saving motive is to earn interest and increase one’s wealth. Geographical factors likely to impact savings and wealth levels thus include variables that proxy for access to financial institutions (e.g. road infrastructure and distance to markets) and the rate of return to capital (e.g. rural versus urban) [57].

A household’s stock of wealth depends also on its the ability to save, which is conditioned largely by the household’s capacity to earn sufficient income as well as local living costs [34-35]. One common view is that specific household and personal attributes, such as low levels of education, lack of competitive labour market skills, or family structure explain a household’s low-income status. Another common view is that low household income is mainly the result of restricted economic opportunities, which may be related to attributes of one’s place of residence, such as urban versus rural, market access, local wage rates, prices, the main occupations in the area (e.g. agriculture, fishing, industry), and the amount and distribution of rainfall during the agricultural period. Restricted opportunities to earn income and generate wealth may also originate from discrimination on the basis of gender, ethnicity or class.

Finally, macro-level variables and interventions have indirect effects on income generation and wealth accumulation through impacts on local geography and individual/household decision parameters. For example, a government’s agricultural price policy strongly influences agricultural incentives and thereby farm household income and wealth. In addition, government investments in education influence individual possibilities to enter remunerative labour markets, earn income and accumulate wealth.

**References**

1. McGregor IA, Wilson RJM: **Specific immunity: acquired in man.** In *Malaria: principles and practice of malariology.* *Volume* 1. Edited by Wernsdorfer WH, McGregor I. Edinburgh; New York: Churchill Livingstone; 1988: 559-619

2. Molineaux L: **The epidemiology of human malaria as an explanation of its distribution, including some implications for its control.** In *Malaria: principles and practice of malariology.* *Volume* 1. Edited by Wernsdorfer WH, McGregor I. Edinburgh; New York: Churchill Livingstone; 1988: 913-998

3. Abdullah S, Adazu K, Masanja H, Diallo D, Hodgson A, Ilboudo-Sanogo E, Nhacolo A, Owusu-Agyei S, Thompson R, Smith T, Binka FN: **Patterns of age-specific mortality in children in endemic areas of sub-Saharan Africa.** *Am J Trop Med Hyg* 2007, **77(Suppl 6):**99–105.

4. McCarthy FD, Wolf H, Wu Y: **Malaria and growth.** In *Book Malaria and growth* (Editor ed.^eds.). City: The World Bank; 2000.

5. Breman JG: **The ears of the hippopotamus: manifestations, determinants, and estimates of the malaria burden.** *Am J Trop Med Hyg* 2001, **64:**1-11.

6. Martens P, Hall L: **Malaria on the move: human population movement and malaria transmission.** *Emerg Infect Dis* 2000, **6:**103-109.

7. Prothero RM: **Forced movements of population and health hazards in tropical Africa.** *Int J Epidemiol* 1994, **23:**657-664.

8. Prothero RM: **Migration and malaria risk.** *Health, Risk & Society* 2001, **3:**19-38.

9. Celli A, Celli-Fraentzel A: *The history of malaria in the Roman Campagna from ancient times.* London,: Bale & Danielsson; 1933.

10. Marques AC: **Human migration and the spread of malaria in Brazil.** *Parasitol Today* 1987**:**166-170.

11. Bloland PB, Williams HA: *Malaria control during mass population movements and natural disasters.* Washington, DC: National Academies Press; 2003.

12. Castro MC, Monte-Mór RL, Sawyer DO, Singer BH: **Malaria Risk on the Amazon Frontier.** *Proc Natl Acad Sci U S A* 2006, **103:**2452-2457.

13. Konradsen F, Amerasinghe P, Hoek Wvd, Amerasinghe F, Perera D, Piyaratne M: **Strong association between house characteristics and malaria vectors in Sri Lanka.** *Am J Trop Med Hyg* 2003, **68:**177-181.

14. Yé Y, Hoshen M, Louis V, Séraphin S, Traoré I, Sauerborn R: **Housing conditions and *Plasmodium falciparum* infection: protective effect of iron-sheet roofed houses.** *Malar J* 2006, **5:8**.

15. Oaks SC, Mitchell VS, Pearson GW, Carpenter CJ: *Malaria: obstacles and opportunities. A report of the Committee for the Study on Malaria Prevention and Control: Status Review and Alternative Strategies, Division of International Health, Institute of Medicine.* Washington, D.C.: National Academy Press; 1991.

16. MacDonald G: *The Epidemiology and Control of Malaria.* London: Oxford University Press; 1957.

17. Okoko BJ: **Acquisition of bed-nets, sleeping-habits, and control of malaria in the Gambia: sociocultural dimension.** *J Health Popul Nutr* 2005, **23:**388-389.

18. Balls MJ, Bødker R, Thomas CJ, Kisinza W, Msangeni HA, Lindsay SW: **Effect of topography on the risk of malaria infection in the Usambara Mountains, Tanzania** *Trans R Soc Trop Med Hyg* 2004, **98:**400-408.

19. Castro MC: **Environmental management of anopheles breeding sites in Dar es Salaam. Phase 1: Inventory of drains in selected areas of Dar es Salaam. Final report.** In *Book Environmental management of anopheles breeding sites in Dar es Salaam. Phase 1: Inventory of drains in selected areas of Dar es Salaam. Final report.* JICA/IMCP/UMCP/HSPH; 2007.

20. Keiser J, Singer BH, Utzinger J: **Reducing the burden of malaria in different eco-epidemiological settings with environmental management: a systematic review.** *Lancet Infect Dis* 2005, **5:**695–708.

21. Keiser J, Utzinger J, Singer BH: **The potential of intermittent irrigation for increasing rice yields, lowering water consumption, reducing methane emissions, and controlling malaria in African rice fields.** *J Am Mosq Control Assoc* 2002, **18:**329-340

22. Boelee E: **Malaria in irrigated agriculture.** *Irrigation and Drainage* 2003, **52:**65-69.

23. Foley JA, DeFries R, Asner GP, Barford C, Bonan G, Carpenter SR, Chapin FS, Coe MT, Daily GC, Gibbs HK, et al: **Global Consequences of Land Use** *Science* 2005, **309:**570-574.

24. Keating J, Macintyre K, Mbogo C, Githeko A, Regens JL, Swalm C, Ndenga B, Steinberg LJ, Kibe L, Githure J, Beier JC: **A geographic sampling strategy for studying relationships between human activity and malaria vectors in urban Africa.** *Am J Trop Med Hyg* 2003, **68:**357-365.

25. Castro MC, Singer BH: **Migration, urbanization and malaria: a comparative analysis of Dar es Salaam, Tanzania and Machadinho, Rondônia, Brazil.** In *Africa on the move: African migration and urbanisation in comparative perspective.* Edited by Tienda M, Findley S, Tollman S, Preston-White E. Johannesburg: Wits University Press; 2006: 280-307

26. Hay SI, Guerra CA, Tatem AJ, Atkinson PM, Snow RW: **Urbanization, malaria transmission and disease burden in Africa.** *Nat Rev Microbiol* 2005, **3:**81-90.

27. Robert V, Macintyre K, Keating J, Trape J-F, Duchemin J-B, Warren M, Beier JC: **Malaria transmission in urban sub-Saharan Africa.** *Am J Trop Med Hyg* 2004, **68:**169-176.

28. Killeen GF, Mckenzie FE, Foy BD, Schieffelin C, Billingsley PF, Beier JC: **A simplified model for predicting malaria entomologic inoculation rates based on entomologic and parasitologic parameters relevant to control.** *Am J Trop Med Hyg* 2000, **62:**535-544.

29. Keiser J, Castro MC, Maltese MF, Bos R, Tanner M, Singer BH, Utzinger J: **Effect of irrigation and large dams on the burden of malaria on global and regional scale.** *Am J Trop Med Hyg* 2005, **72:**392-406.

30. Lerer LB, Scudder T: **Health impacts of large dams.** *Environ Impact Asses* 1999, **19:**113-123.

31. World Health Organization: **Human Health and Dams.** In *Book Human Health and Dams* (Editor ed.^eds.). City: The World Health Organization's submission to the World Commission on Dams (WCD). 2000.

32. Keynes JM: *The general theory of employment, interest and money.* London: Macmillan and co., limited; 1936.

33. Modigliani F: **Life cycle, individual thrift, and the wealth of nations.** *Am Econ Rev* 1986, **76:**297-313.

34. Rank MR, Yoon H, Hirschl TA: **American poverty as a structural failing: evidence and arguments.** *Journal of Sociology and Social Welfare* 2003, **30:**3-29.

35. Schiller BR: *The Economics of Poverty and Discrimination.* Englewood Cliffs, NJ: Prentice Hall; 1995.
